# It is no longer the time to disregard thyroid metastases from breast cancer: a case report and review of the literature

**DOI:** 10.1186/s12885-018-4054-x

**Published:** 2018-02-06

**Authors:** Matilde Pensabene, Brigida Stanzione, Ivana Cerillo, Giuseppe Ciancia, Immacolata Cozzolino, Raffaella Ruocco, Caterina Condello, Giuseppe Di Lorenzo, Mario Giuliano, Valeria Forestieri, Grazia Arpino, Sabino De Placido, Rossella Lauria

**Affiliations:** 1 Department of Clinical Medicine and Surgery, University of Naples “Federico II”, via Sergio Pansini, 80131 Naples, Italy; 2 Department of Medical Oncology, National Cancer Institute, Aviano, PN Italy; 3 Department of Advanced Biomedical Sciences, Pathology Unit, University of Naples “Federico II”, via Sergio Pansini, 80131 Naples, Italy

**Keywords:** Breast cancer, Metastases to thyroid, Lobular breast cancer, Goitre

## Abstract

**Background:**

Metastases to the thyroid gland are more frequent than previously thought, although most of them are occult or not clinically relevant. Overall, only 42 cases of metastases to thyroid from breast cancer have been reported thus far. Here we report the case of a patient with breast cancer metastatic to the thyroid. We also review the 42 previously reported cases (published between 1962 and 2012). This is the first review about metastases to thyroid gland from breast cancer.

**Case presentation:**

A 64-year-old woman of Caucasian origin was diagnosed with a lobular invasive carcinoma of the breast (luminal A, stage II). She received adjuvant chemotherapy, followed by endocrine therapy. During follow-up, fine-needle cytology of a thyroid nodule revealed malignant cells that were estrogen-positive, which suggested a diagnosis of metastases to the thyroid. Imaging did not reveal any other metastatic site and showed only enlargement of the left thyroid lobe and an inhomogeneous pattern of colloid and cystic degeneration and calcifications. The patient underwent left hemithyroidectomy. Histology of thyroid tissue showed a colloid goitre containing dispersed small atypical neoplastic cells with eccentric nuclei. Immunohistochemistry showed cytokeratin-19 and oestrogen receptor, but not tireoglobulin, e-cadherin or cytokeratin-7, thereby confirming metastases from a lobular breast carcinoma. Hormonal treatment is ongoing.

**Conclusion:**

This case report and first review of the literature on metastases to thyroid from breast cancer highlight the importance of a correct early diagnostic work-up in such cases. Indeed, a primary lesion should be distinguished from metastases given the different treatment protocol related to primary cancer and the clinical impact on prognosis.

## Background

Metastatic cancer in the thyroid is uncommon and accounts for about 1.4–3% of malignant solid tumours [1–6]. The most frequent primary cancers are renal cell (48.1%), colorectal (10.4%), lung (8.3%) and breast (7.8%) cancers, and soft tissue sarcoma (4.0%) [1]. Large series reported also lymphoma as primary cancer or metastases from lung cancer other than usual epithelial thyroid cancers [7]. Also parotid cancer and melanoma have been reported as primary cancers [8, 9]. Formerly, metastases to the thyroid were usually identified at autopsy [9]. Thanks to the advent of more accurate diagnostic methods, it is now possible to clinically diagnose metastases to the thyroid, and initiate timely surgical and systemic treatment thereby improving outcome [2].

## Case report

We report the case of a 64-year-old woman who was diagnosed with left breast cancer in June 2011. The comorbidities were multinodular goitre of the thyroid gland, obstructive pulmonary disease (i.e. emphysema), left atrial enlargement with severe pulmonary hypertension, carotid stenosis (60–65%) and severe obesity. Clinical staging by chest X-ray, abdominal ultrasound and bone scan was negative, except for CEA = 10.7 (normal value < 5). The patient underwent wide excision of the breast lesion with axillary node dissection. Histology revealed a G2 invasive lobular carcinoma with lymph node metastasis, stage pT1cN3 (21/24 nodes), with positive staining for oestrogen receptor (ER 90%) and progesterone receptor (PgR 80%), a very low proliferation index (Ki67 < 10%), and without amplification of HER2-neu, suggesting a luminal A phenotype. Adjuvant chemotherapy with docetaxel and cyclofosfamide was administered, followed by endocrine therapy with aromatase inhibitor. At the first follow-up, clinical examination showed enlarged thyroid lobes with a multinodular structure; a thyroid function test was normal. Ultrasound revealed an enlarged thyroid with retrosternal development; the glandular structure was finely inhomogeneous in the right lobe, and the entire left lobe was occupied by a large nodule that had a mixed echo-pattern and areas of cystic and colloid degeneration and calcifications.

**Fig. 1:**
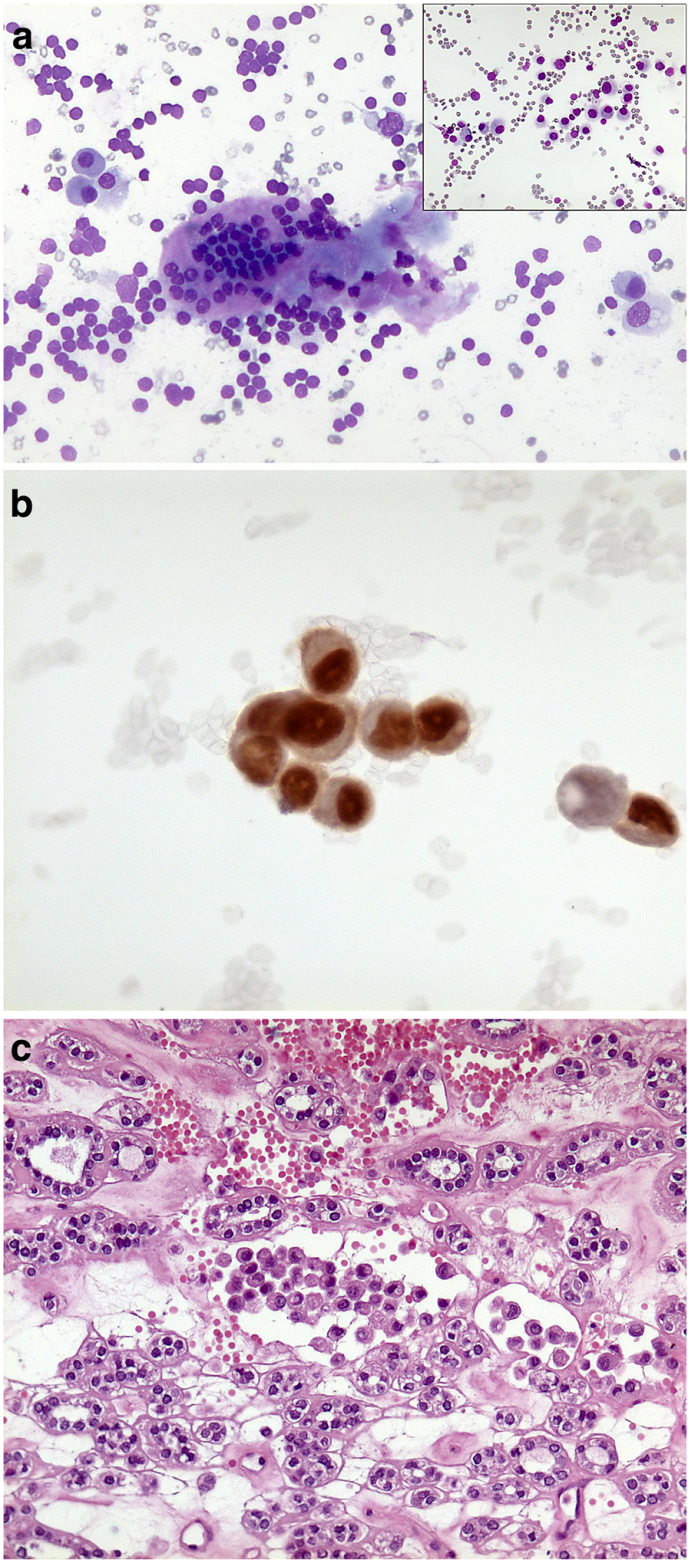
**a** Cytology of thyroid metastases and plasmacytoid-like aspect (inset). **b** Immunocytochemistry with positive estrogen receptor staining. **c** Histology of thyroid metastases

**Fig. 2:**
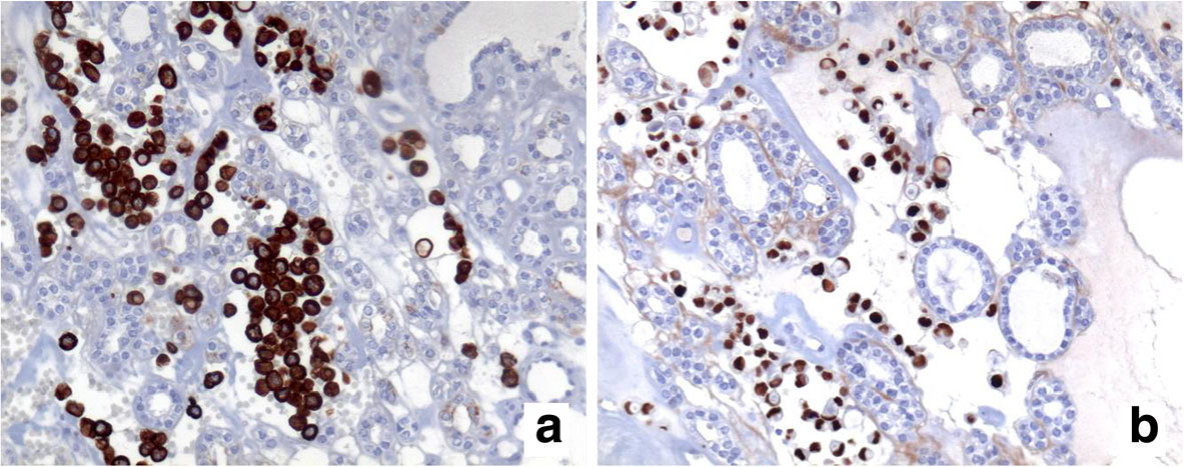
Immunohistochemistry showing cytokeratin 19 (**a**) and estrogen receptor (**b**)

Fine needle cytology was performed with a 23-G needle without suction. Smears taken from both lobes were Diff-Quik-stained and evaluated on-site. Cellularity was deemed to be satisfactory, and additional smears were taken and alcohol-fixed for Papanicolaou stain and for ancillary techniques. The right lobe smear was moderately cellular and showed only colloid and benign thyrocytes. Conversely, the left lobe smears revealed a second cellular population in a colloidal background mixed with small groups of benign thyrocytes (Fig. 1A). This second population was constituted by small cells, dispersed or aggregated into small, loosely formed groups; individual cells had a plasmacytoid-like aspect (Fig. 1A, inset) and occasionally a secretion vacuole. Given the patient’s history, we decided to use two smears fixed in alcohol to evaluate oestrogen receptor expression in the second cell population. Immunocytochemistry revealed positive oestrogen receptor staining (Fig. 1B), which suggested a diagnosis of metastases of the breast cancer to the thyroid. Positron emission tomography and total body tomography did not reveal other metastatic sites, and showed only enlargement of the left thyroid lobe and an inhomogeneous pattern of colloid and cystic degeneration and calcifications. Therefore, the patient underwent left hemithyroidectomy in February 2012. Histology revealed thyroid tissue with a colloid goitre containing dispersed neoplastic cells constituted by small atypical cells with eccentric nuclei (Fig. 1C). Immunohistochemistry revealed cytokeratin-19 and oestrogen receptor (Fig. 2 a, b), but not tireoglobulin, e-cadherin or cytokeratin-7, thereby suggesting metastases from a lobular breast carcinoma. Thirty-two months after hemithyroidectomy, the patient is alive, although in May 2014, there was evidence of recurrence in bone. Hormonal treatment with fulvestrant is ongoing. She died in July 2015.

## Discussion and conclusions

### Methods of review

We searched PubMed to identify studies about metastases to thyroid from different primary tumours, including breast cancer. Searches were made using the terms ‘breast cancer’ and ‘metastases to thyroid’, with no limitation of language, publication date, or journal of publication. Eighteen articles were eligible according to our criteria; these were published between 1962 and 2012. Given the rarity of metastases to the thyroid and the limited number of reported cases, we performed only a descriptive analysis.

### Epidemiology

Metastases to the thyroid gland are rare, but not as rare as previously thought. This is not surprising because the thyroid gland is the second most richly arterialized organ in the body. The probability of finding metastases in the thyroid gland depends on the method of investigation [3]. Large autopsy studies found that the incidence of thyroid metastases in patients with a history of cancer ranges from 1.9% to 24% [1, 3, 8, 9]. Two of these studies suggested that thyroid metastases are more common than primary thyroid cancer [1, 3]. On the other hand, the incidence of thyroid metastases in clinical and surgical series was 3% [4]. Reports of thyroid metastases have increased in recent years consequent to more sophisticated diagnostic methods, i.e. fine needle cytology and proton emission tomography with 18F–fluorodeoxyglucose [1, 10].

In autopsy series, breast cancer, lung cancer and melanoma were found to be the most frequent malignancies to metastasize to the thyroid [11]. Clinical and surgical series of patients showed that breast carcinoma is the second most common primary tumour to result in symptomatic thyroid metastases, the first being clear cell renal cancer [5, 11, 12].

### Clinical and pathological presentation

The characteristics of breast cancer patients with thyroid metastases are reported in Table 1. We analyzed sex, age at diagnosis, histology, primary treatment, treatment failure, time between primary diagnosis and thyroid metastases and follow-up in 42 women with thyroid metastases from breast cancer reported between 1962 and 2012 [1–20]. The development of metastases of the thyroid gland does not appear to be age-dependent, and seems to be more frequent in women [1]. The median age at diagnosis of metastases to the thyroid gland is 51 years (range: 22–83 years) [9, 12]. Time-to-detection and time from presentation to death differ among reports. The former ranges from 2 months to more than 15 years after the diagnosis of the primary cancer [3, 5], and the latter from 1 to 34 months [13]. In one case, thyroid metastases were synchronous to primary breast cancer [10].

**Table 1:** Characteristics of breast cancer patients with metastases to thyroid in a clinical series

Authors	Study yrs	No of pts	Sex	Age	Histology	Primary treatment	Treatment failure Local Distant		Time between BC diagnosis and thyroid metastasis	Follow-up
Shimaoka K et al. 1962 [9]	1955	1	F	44	carcinoma	CT	no	LN, bone, liver, skin, CNS	5 yrs	4 months
Wychulis AR et al., 1964 [14]	1907–1962	3	F	38	adenocarcinoma	S	no	lung, bone	13 yrs	4 yrs. died of the disease
			F	51	adenocarcinoma	S + RT	Yes	NR	3 yrs	5 months lost to FU
			F	51	adenocarcinoma	S + RT	NR	NR	Synchronous (0 months)	1 yr. lost to FU
Harcourt-Webster JN, 1965 [15]		2	F	56	adenocarcinoma	S	no	liver, bone		
			F	50	adenocarcinoma	S				
Pillay SP et al., 1977 [29]	1974–1976	2	F	58	adenocarcinoma		no	yes		
			F	58						
Nakhjavani MK et al. 1997 [5]	1985–1994	7	F	67	adenocarcinoma	NR	NR	NR	from 2 months to 22 yrs	3 patients < 3 months 4 pts. 3–23 months
Chung SY et al., 2001 [17]	1995–2000	6	F	49	NR		No	lung, bone		
			F	61				lung		
			F	51				lung, bone, liver		
			F	32				lung, liver		
			F F	22 33				bone, peritoneum lung		
De Ridder M et al. 2003 [13]	1982–2002	1			NR	HT	No	no	0 months	Died after 2 years for disseminated disease
Loo CK, Burchett IJ, 2003 [18]		1	F	52	neuroendocrine carcinoma	HT	No	bone	8 yrs	
Wood K et al., 2004 [3]	1985–2002	1	F	72	adenocarcinoma		No	no	15 yrs	36 months alive
Owens CL et al., 2005 [19]	2005	1	F	64	NR		No	shoulder subcutaneous nodule, liver	5 yrs	
Kim TY et al. 2005 [10]	1997–2003	5	F	36	ductal	CT	No	Neck LN, lung	18 months	6 months alive
			F	34	ductal	CT		lung, ax LN, scal	25 months	17 months alive
			F	44	ductal	CT		Nil	37 months	4 months alive
			F	55	ductal	CT		lung, ax LN, neck	68 months	26 months alive
			F	45	ductal	CT		Neck LN, lung, bone	85 months	8 months alive
Gerges AS et al. 2006 [8]	1989–2004	1	F	45	ductal	S + CT + RT	No	bone	63 months	37 months alive post-thyroidectomy
Cichon A et al., 2006 [2]	1984–2003	1	F	50		S	No	no	10 yrs	24 months alive
Molina Garrido MJ et al., 2006 [11]	2005	1	F	43	NR	mastectomy, lymphadenectomy CT + RT	No	lung, liver	30 months	1 month
Papi G et al., 2007 [6]	1993–2003	5	F		ductal		No	no		5 yrs. 1 alive
			F		NR					
			F		NR					
			F		NR					
			F		NR					
Calzolari F et al., 2008 [4]	1995–2005	1	F		NR		No	no		60 months died of the r disease
Saber A et al. 2007 [20]	NR	1	F		NR	S + CT	NR	no	60 (range: 13–135 months)	
Egaña N et al., 2012 [12]	2007	1	F	83	NR	mastectomy, lymphadenectomy HT	No	bone, liver and pelvic mass	3 yrs	1 month
Current report	2011	1	F	64	ILC	S + CT + RT	No	bone	6 months	18 months alive

*F* female, *CT* chemotherapy, *FU* follow-up, *LN* nodes, *yrs.* years, *HT* hormonal treatment, *S* surgery, *RT* radiotherapy, *NR* not reported, *ILC* infiltrating lobular carcinoma, *CNS* Central Nervous System

As shown in Table 1, the clinical presentation of thyroid metastases is very heterogeneous. They are clinically evident only in a minority of patients and are frequently found incidentally during postoperative follow-up by ultrasonography. Thyroid metastases usually present in the context of widespread metastatic disease, and manifestations in the thyroid are not clinically significant. On the other hand, when thyroid metastases are the first presentation of recurrent disease, they usually appear as a palpable neck mass or, albeit less often, with dysphagia, massive tracheal involvement or dysphonia. Often, patient presented with painless neck mass [21].

In the reports containing histological information, breast cancer is generically referred to as “adenocarcinoma” [3, 14, 15]. Where indicated, the most prevalent breast cancer is ductal infiltrating carcinoma [6, 8, 10], reported in seven cases, while invasive lobular carcinoma was reported in only our case and in a case described by Egana et al. [12]. Not all the studies reviewed reported the site of recurrence. In six cases. The thyroid was the first and only site of recurrence [2–4, 6, 13]. As shown in Table 1, other sites of widespread disease were lung, liver and bone. In eleven cases, breast cancers recurred in different sites, but the metastatic site other than thyroid was not reported [5, 14, 16].

### Diagnostic work-up

**Fig. 3:**
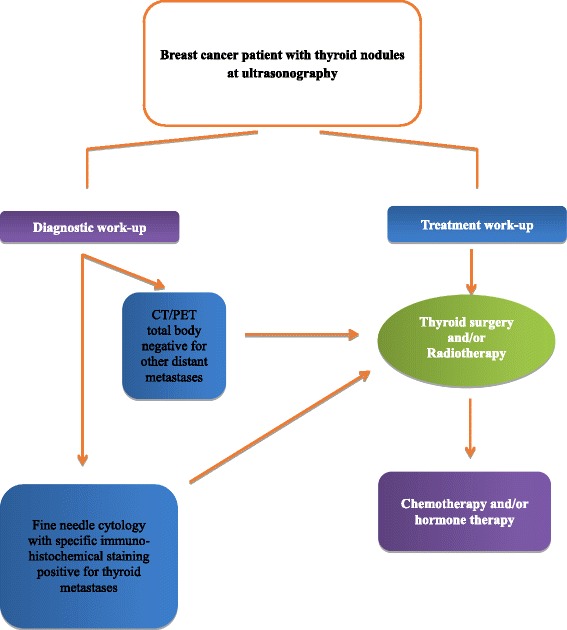
Diagnostic work-up and treatment of patient with thyroid metastases from breast cancer

Figure 3 shows a diagnostic and therapeutic work-flow of patients with suspected metastases to the thyroid from breast cancer. Thyroid nodules in a patient with a history of malignancy should be investigated particularly if they appear many years after the primary tumour. In fact, a malignant thyroid nodule in such patients is much more likely to be metastatic than a new primary tumour. Thyroid metastases cannot be differentiated from a primary thyroid cancer based on clinical features, therefore fine needle cytology must be included in the diagnostic work-up, particularly given its low morbidity and cost, and its high negative predictive value [22–24]. Cytology generally shows abundant cellularity and the cells may be typical of the original site, especially when specific immunohistochemical stains are used. Negative staining with anti-thyroglobulin and anti-calcitonin antibodies would favour a diagnosis of metastatic tumour.

When papillary and follicular carcinomas have a complex pathological pattern, cytology alone cannot reveal the origin of the metastatic tumour. The diagnosis is particularly difficult in case of less common primary thyroid cancers such as small cell, giant cell and spindle cell carcinomas, anaplastic cancer and the clear cell variant of follicular carcinoma. Therefore, biopsy is needed to reach a definitive diagnosis. In all the series reported so far, the diagnosis was confirmed cytologically and histologically [1]. Regarding the cytological differential diagnosis, a non-cohesive cell population and a plasmacytoid-like aspect can mimic a medullary carcinoma of the thyroid. In medullary carcinoma, cytological smears are usually more cellular in a background without colloid, and frequently contain amorphous material consistent with amyloid. Tumour cells are predominantly isolated, but clusters and rosettes may also be seen. Cells have a plasmocytoid appearance and are uniform in size and shape with moderate or abundant, finely granular cytoplasm and eccentrically placed nuclei. Many smears show large cells with nuclear megaly, and bi-nucleated and multinucleated cells. These aspects were not observed in our patient. Indeed, in our case, the history of breast cancer, the absence of typical findings of papillary or follicular carcinoma, positive staining of oestrogen and progesterone receptors, negative staining of both thyroglobin and calcitonin, and the histological pattern of the primary and metastatic tumour enabled us to establish a diagnosis of metastases from thyroid. In particular, the following immunocytochemical markers were analyzed: cytokeratin 7, cytokeratin 19, E-cadherin, CD34, besides estrogen and progesterone receptors.

When thyroid metastases are found, it is important to re-evaluate the diagnosis of the primary tumour and search for other metastatic sites. Because breast cancer has been associated with thyroid disease and because thyroid nodules are more frequently found in women [25], it is important to examine the thyroid during breast ultrasonography. Ultrasonography generally shows lesions with irregular shape and inhomogeneus [21]. At computer tomography, metastases to thyroid are hypodense; while they look iso-hyperintense in comparison to the normal thyroid tissue at magnetic resonance imaging [21]. Thyroid examination in the work-up of patients with breast cancer and goitre or nodules can reveal thyroid metastases in an early phase. The oncologist should consider that thyroid functional tests and radioiodine uptake are normal in most patients [1].

### Management of thyroid metastases

The treatment of thyroid metastases depends on the site of the primary tumour, presence of other metastases and symptoms caused by the thyroid mass. Surgery is considered the gold standard treatment for thyroid metastases. Radical treatment of an isolated metastasis to the thyroid can be curative, and an aggressive surgical approach has been recommended especially in case of slow growing tumours such as breast or kidney carcinomas [3]. The extension of surgical resection does not seem to significantly impact on survival. In fact, no significant differences in survival were found between total thyroidectomy and conservative surgery [5]. Surgical treatment of isolated metastasis may prolong survival [5]. However, more data are necessary regarding the best surgical approach in patients with a single thyroid metastasis [4].

Patients with single metastases to the thyroid should be treated surgically, whereas patients with multiple metastases in different organs should be treated with a hormonal or chemotherapeutic approach in accordance with international advanced breast cancer guidelines for extensive disease [26]. For patients with metastatic sites other than thyroid, surgery is generally performed to reduce pressure, which causes discomfort, and to avoid airway obstruction and skin ulceration [4].

Data concerning radiotherapy or chemotherapy for metastatic disease are fragmentary and limited. Wychulis et al. [14] reported that radiotherapy relieved symptoms, and should thus be considered an option, particularly in patients with high anaesthetic risk and a clinical condition that precludes surgery. Radioactive iodine 131I has not been found to be effective [27].

### Prognosis

Reports of thyroid metastases span over more than four decades. It is not feasible to make a global evaluation of the outcome of patients because of the heterogeneity of treatments and some systemic therapies that have become obsolete. However, numerous case reports suggest that metastases to the thyroid gland are associated with a poor prognosis [28]. Multifocal metastases seem to adversely affect prognosis. Indeed, a significantly worst survival has been reported in patients with multiple foci. Survival after surgical treatment is variable with some patients succumbing to metastatic disease within a few months, while others have a long-term survival. Data on prognosis cannot be extrapolated also in view of the many advances made in systemic treatment and in the identification of distinctive biological features of breast cancer from the first published case in 1962 until now. In particular, the introduction of taxanes as well as targeted therapies, such as trastuzumab and pertuzumab for HER2-positive tumours and bevacizumab for HER2-negative tumours, which have had an enormous positive effect on outcome. There are no case reports that describe the type of systemic treatment used, and patients were often treated with surgery, so that outcomes cannot be extrapolated to define the prognosis of breast cancer with metastases to the thyroid gland.

### Conclusions

In this review and case report we examine aspects of breast cancer metastases to the thyroid going from the diagnostic workup to the treatment. Metastases to the thyroid gland can present many years after treatment of a distant primary tumour; however, in a patient with a history of malignancy, a neoplastic thyroid nodule is more likely to be a metastasis than a new thyroid malignancy. Fine-needle aspiration biopsy of the thyroid gland should be performed in patients with breast cancer and a nodular goitre, even in the absence of clinical signs of metastatic disease. Biological features are important for treatment decision-making. Given the availability of targeted biological therapies, i.e. transtuzumab, pertuzumab and bevacizumab, that modify the natural history of metastatic breast cancer, it is no longer the time to disregard the thyroid metastases from breast cancer and other primary malignancies.

The history of our patient suggested thyroid goitre and showed no clinical feature suggestive of metastasis. Our case report highlights that metastases, also such an unusual site as the thyroid, should be considered in patients diagnosed with breast cancer. Studies dealing with thyroid metastases are very heterogeneous in terms of the primary cancer, which makes it difficult to evaluate the impact of thyroid metastases on prognosis. Most patients with thyroid metastases have widespread metastatic disease but occasionally the thyroid may be the only site of disease. Although therapy of metastatic malignancies is often considered to be palliative, aggressive surgical treatment in isolated cases may be curative and may benefit survival. This highlights the importance of early recognition and management of thyroid metastases in prolonging survival in some patients and in preventing the onset of life-threatening complications.
